# Antenatal Hypoxia Induces Programming of Reduced Arterial Blood Pressure Response in Female Rat Offspring: Role of Ovarian Function

**DOI:** 10.1371/journal.pone.0098743

**Published:** 2014-06-06

**Authors:** DaLiao Xiao, Xiaohui Huang, Qin Xue, Lubo Zhang

**Affiliations:** Center for Perinatal Biology, Division of Pharmacology, Department of Basic Sciences, Loma Linda University School of Medicine, Loma Linda, California, United States of America; University of Southampton, United Kingdom

## Abstract

In utero exposure to adverse environmental factors increases the risk of cardiovascular disease in adulthood. The present study tested the hypothesis that antenatal hypoxia causes a gender-dependent programming of altered arterial blood pressure response (BP) in adult offspring. Time-dated pregnant rats were divided into normoxic and hypoxic (10.5% O_2_ from days 15 to 21 of gestation) groups. The experiments were conducted in adult offspring. Antenatal hypoxia caused intrauterine growth restriction, and resulted in a gender-dependent increase Angiotensin II (Ang II)-induced BP response in male offspring, but significant decrease in BP response in female offspring. The baroreflex sensitivity was not significantly altered. Consistent with the reduced blood pressure response, antenatal hypoxia significantly decreased Ang II-induced arterial vasoconstriction in female offspring. Ovariectomy had no significant effect in control animals, but significantly increased Ang II-induced maximal BP response in prenatally hypoxic animals and eliminated the difference of BP response between the two groups. Estrogen replacement in ovariectomized animals significantly decreased the BP response to angiotensin II I only in control, but not in hypoxic animals. The result suggests complex programming mechanisms of antenatal hypoxia in regulation of ovary function. Hypoxia-mediated ovary dysfunction results in the phenotype of reduced vascular contractility and BP response in female adult offspring.

## Introduction

Adverse *in utero* environment during a critical period of fetal development results in fetal adaptive changes that may lead to an increased risk of cardiovascular disease in postnatal life [1]–[4]. Although many studies have shown that low birth weight individuals are more likely to develop higher arterial pressure compared with those of normal birth weight, in some clinical and experimental studies the magnitude of the effect is variable in different animal models and populations [5]–[7]. Pausova et al [8] reported that *in utero* adverse environment enhanced blood pressure in spontaneously hypertensive rats but not in normotensive Brown Norway rats. In addition, a number of studies have shown that adverse environment-induced low birth weight is associated with low blood pressure in offspring [9]–[11]. Furthermore, the sex difference has been reported in several animal models of fetal programming of cardiovascular responses [12]–[15]. Ojeda at al [14] showed that fetal programming of hypertension was reversed by estrogen in adult female offspring. These studies suggest the complexity of fetal programming of cardiovascular responses that may be determined by the interaction of intrauterine environment and sex steroid hormones in the postnatal development.

Hypoxia during gestation is one of the most common intrauterine stresses that may alter fetal growth and development. Previous studies have suggested a possible link between maternal hypoxia and increased risk of cardiovascular dysfunction in offspring [16]–[22]. Our recent studies in rats have demonstrated that maternal hypoxia results in increased cardiac vulnerability to ischemia and reperfusion injury in adult male, but not female offspring [12], [16]. Although previous studies have shown that prenatal hypoxia increases blood pressure response in adult male offspring [18], and alters postnatal vascular function [13], [20], to our knowledge, the effect of antenatal hypoxia on programming of blood pressure response in adult female offspring has not been determined. Therefore, the present study tested the hypothesis that gestational hypoxia induces programming of altered blood pressure response in sex-specific manner in female offspring. Given that ovarian sex steroid hormones play an important role in regulating cardiovascular responses and ovarian function may be altered by adverse *in utero* environment [23]–[26], we also determined the role of ovary and sex hormones in antenatal hypoxia-mediated programming of blood pressure response in female offspring.

## Methods

### Experimental animals

All procedures and protocols were approved by the Institutional Animal Care and Use Committee of Loma Linda University (IACUC#8110036) and followed the guidelines by the National Institutes of Health Guide for the Care and Use of Laboratory Animals. Time-dated pregnant Sprague-Dawley rats which were non previous breeders were purchased from Charles River Laboratories (Partage, MI). The rats were randomly divided into 2 groups: (1) normoxic control (n = 20) and (2) hypoxic (n = 18) treatment of 10.5% O_2_ from days 15 to 21 of gestation, as reported previously [27]. Water and food were provided as desired. After hypoxic treatment, six control and five hypoxic pregnant rats were sacrificed, and the fetuses (21 days of gestation) were isolated for body weight measurement. The other pregnant rats (14 control and 13 hypoxic treated pregnant rats) were allowed to deliver at term. A total of 153 pups from the control and 148 pups from hypoxic pregnant rats were delivered. Our previous studies [16], [28] and current studies did not show any significant differences in litter size following the hypoxic insult. Therefore, the litter size was intact as nature in each dam and all of the newborn pups were kept with their mothers until weaning. At weaning (3-weeks age), the male and female offspring were separated. There were eight groups of offspring (control males, hypoxic males, control intact females, control-OVX females, control-OVX-estradiol females, hypoxic intact females, and hypoxic –OVX-estradiol females) in total of 160 offspring (20 in each group) kept for following BP measurement, contractile function and other experiments. The offspring of one dam were split between experimental groups.

### Ovariectomy and estrogen replacement

Previous studies have suggested that the puberty age of female Sprague Dawley rat is about 55 days-old [14], [29]. Therefore, Ovariectomy (OVX) was performed at 8-week-old females as previously described [14], [30]. Briefly, animals were anesthetized with 75 mg/kg ketamine and 5 mg/kg xylazine injected intramuscularly, and adequate anesthesia was determined by loss of pedal withdrawal reflex. The skin was opened by a ventral midline incision and ovarian vessels were tied off, and ovaries were removed (OVX group). The sham operation had a ventral midline incision, and ovaries were visualized but not removed. Seven days after ovariectomy surgery, 17β-estradiol valerate minipellets (7.5 mg for 90-day release, Innovative Research of American) were implanted in half of animals for estrogen replacement in the physiological range [14], [30]. Starting at 3 month old of age, the offspring were been conducting functional experiments.

### Measurement of estrogen (E_2_) Levels

Plasma E_2_ levels were determined with a commercially available estradiol ELISA kit (BioQuant BQ180S).

### Measurement of arterial blood pressure

Offspring at 3-month-old was implanted with a catheter in the femoral artery to record arterial blood pressure and heart rate, and a catheter in the femoral vein for drug administration, as described previously [30], [31]. Two days after recovery from surgery, blood pressure and heart rate were measured continuously in conscious animals. After the baseline recording for 60 minutes, animals received a bolus injection of angiotensin II (Ang II) (300 ng/kg, 1 ml/kg, i.v.), and blood pressure was recorded for another 60 minutes, as described previously [31], [32]. The data of systolic, diastolic, and mean arterial blood pressure, and HR were recorded continuously throughout each study with data acquisition software (Powerlab 16/SP and Chart version 4, ADInstruments). The baroreflex sensitivity was calculated as slope of Ang II-induced heart rate/MAP (bpm/mmHg) from all of the time points including the rising and declining BP data points.

### Measurement of arterial contractions

Aortas were isolated from animals, cut into 4-mm rings and mounted in 10-ml tissue baths containing modified Krebs solution equilibrated with a mixture of 95% O_2_ and 5% CO_2._ Isometric tensions were measured at 37°C, as described previously [30], [31]. After 60 minutes of equilibration, each ring was stretched to the optimal resting tension as determined by the tension developed in response to 120 mmol/L KCl added at each stretch level. Ang II-induced concentration-dependent contractions were obtained by cumulative additions of the agonist in approximate one-half log increments.

### Statistical analysis

Concentration-response curves were analyzed by computer-assisted nonlinear regression to fit the data using GraphPad Prism (GraphPad Software, San Diego, CA) to obtain pD_2_ (-log EC_50_) and the maximum response (E_max_). Results were expressed as mean ± SEM. Statistical significance (*P*<0.05) was determined by analysis of variance (ANOVA) followed by Neuman-Keuls post hoc testing or Student's *t* test, where appropriate.

## Results

### Effect of antenatal hypoxia on body weight and litter size

As shown in table 1, antenatal hypoxia significantly decreased body weight of near-term (21-day) male fetuses and female fetuses, which is consistent with our previous reports in the same animal model that maternal hypoxia decreases fetal birth body weights and 7-days neonatal body weights [16], [28]. However, there was no significant difference in body weight of 3-month-old adult male and female offspring between control and hypoxic groups, suggesting a catch-up growth in hypoxic animals. The litter size was not significant differences between the control and hypoxic treatment groups (11.75±0.42 vs 11.83±0.32 pups/dam, n = 20 for control, n = 18 for hypoxia, P>0.05). In addition, the ratio of males to females was also not significant difference between the control and hypoxic treatment groups (1.35±0.18 vs 1.09±0.17, P>0.05).

**Table 1:** Effect of Hypoxia on Body Weight and Basal Heart Rate.

Animal group		Fetal BW (g)	n	Adult BW (g)	n	HR (beat/min)	n
Male	Control	4.461±0.046	48	637.5±22.4	10	340.2±13.0	9
	Hypoxia	3.342±0.077*	32	597.5±25.6	10	334.8±9.2	9
Female	C-sham	4.13±0.057	33	282.9±6.5	9	359.0±7.8	9
	H-sham	3.27±0.074*	33	276.5±6.8	10	370.9±11.0	9
	C-OVX			352.5±8.1^a^	9	355.2±10.9	9
	H-OVX			349.4±12.4^a^	9	361.9±14.1	8
	C-OVX+E2			227.5±4.4^a^ ^,b^	10	333.4±8.5	9
	H-OVX+E2			220.6±7.4^a^ ^,b^	9	349.5±11.2	8

Note: BW, body weight; HR, heart rate; *P < 0.05, Hypoxia (H) vs. control(C);

a P<0.05, vs. sham; ^b^P<0.05, vs. OVX.

### Effect of antenatal hypoxia on Ang II-induced blood pressure response

**Figure 1:**
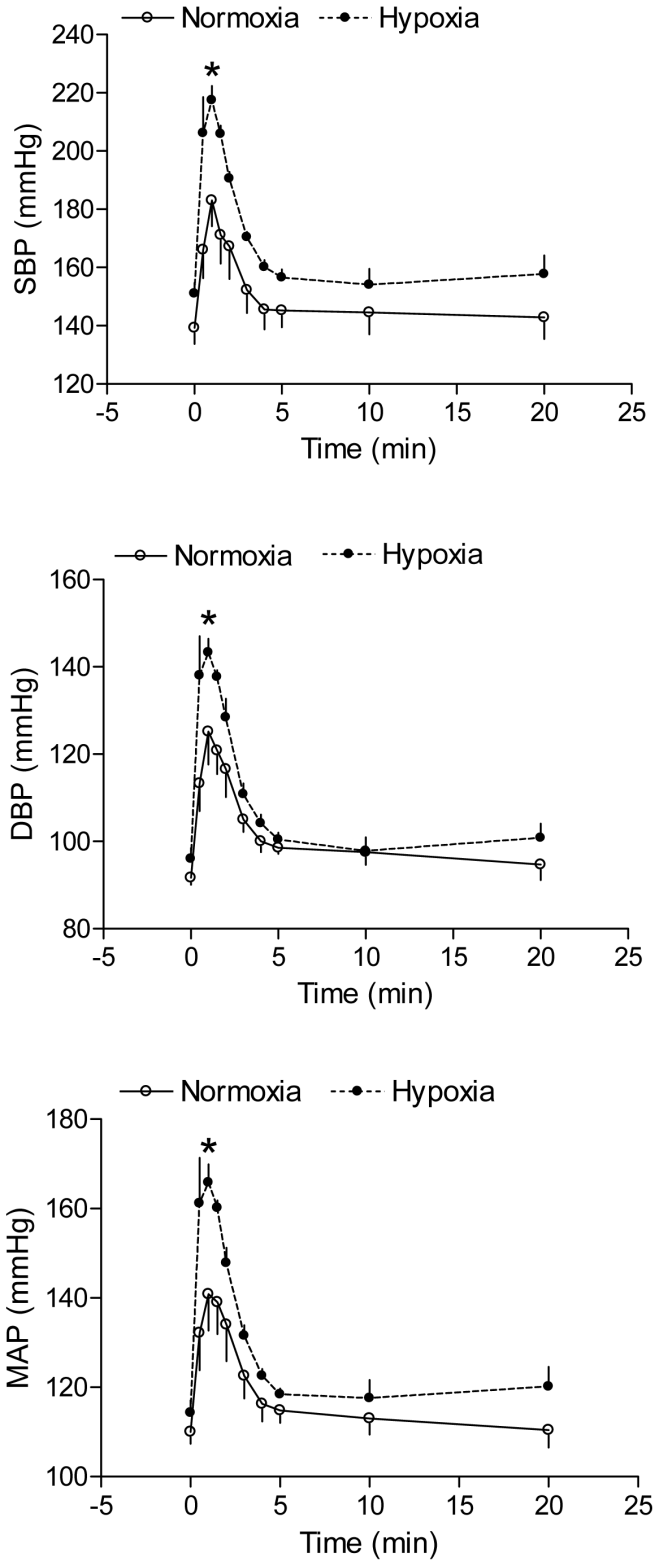
Effect of antenatal hypoxia on angiotensin II-induced blood pressure response in male offspring. Systolic (SBP), diastolic (DBP), and mean (MAP) arterial blood pressure responses to angiotensin II (300 ng/kg; 1 ml/kg, *i.v.*) were measured in 3-month-old male offspring that had been exposed *in utero* to normoxia or hypoxia. Data are means ± SEM, n = 7. * P<0.05, hypoxia *vs.* normoxia.

**Figure 2:**
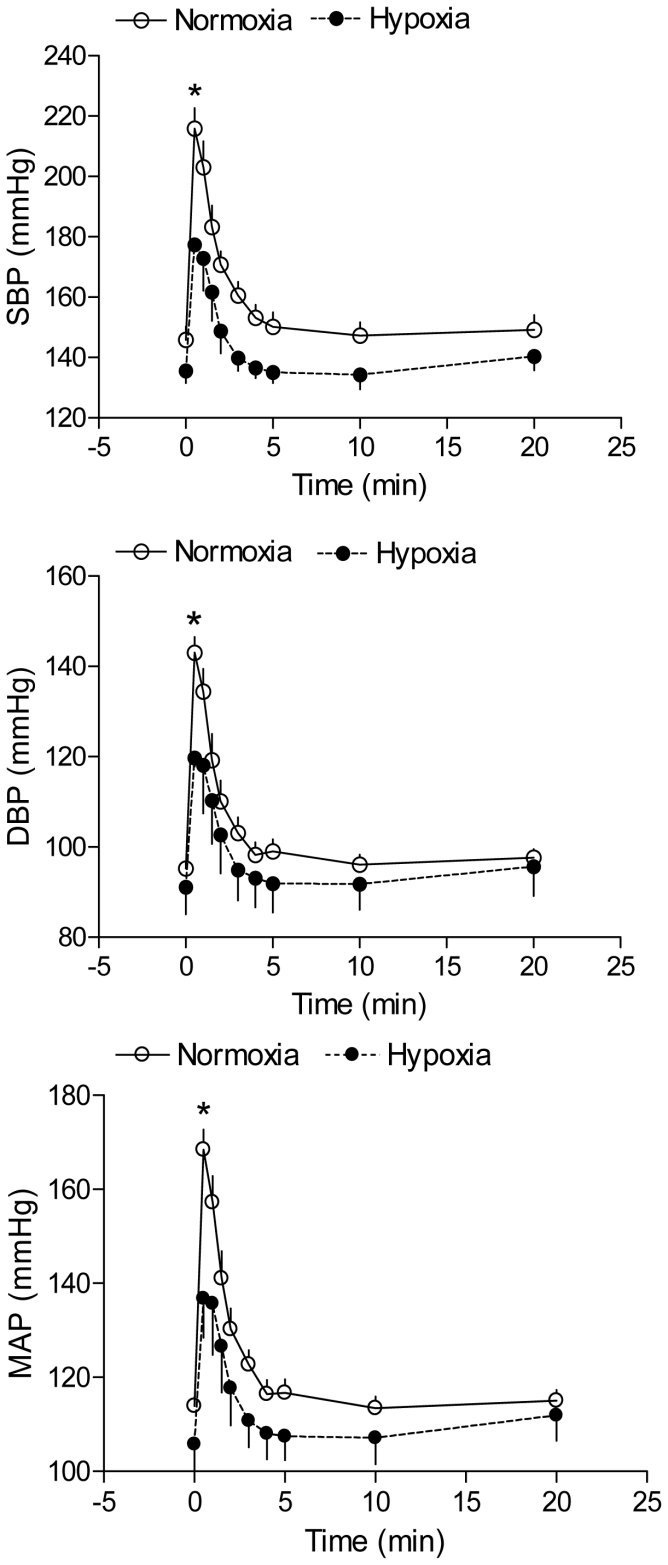
Effect of antenatal hypoxia on angiotensin II-induced blood pressure response in female offspring. Systolic (SBP), diastolic (DBP), and mean (MAP) arterial blood pressure responses to angiotensin II (300 ng/kg; 1 ml/kg, *i.v.*) were measured in 3-month-old female offspring that had been exposed *in utero* to normoxia or hypoxia. Data are means ± SEM, n = 9. * P<0.05, hypoxia *vs.* normoxia.

**Figure 3:**
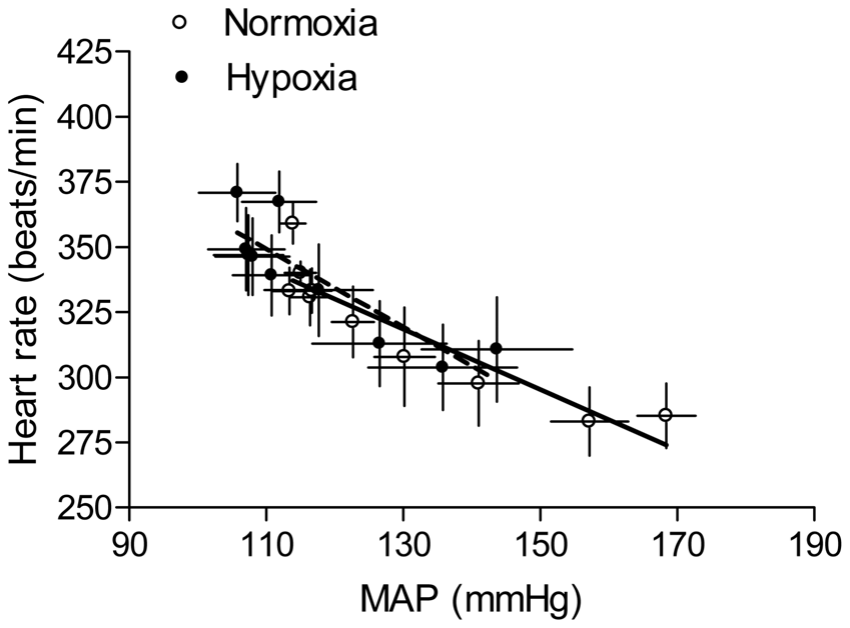
Effect of antenatal hypoxia on the baroreflex sensitivity in female offspring. Heart rate and mean arterial blood pressure (MAP) responses to angiotensin II (300 ng/kg; 1 ml/kg, *i.v.*) were measured in 3-month-old female offspring that had been exposed *in utero* to normoxia or hypoxia. The baroreflex sensitivity was determined as the slope of heart rate/MAP (bpm/mmHg). Data are means ± SEM, n = 9.

**Figure 4:**
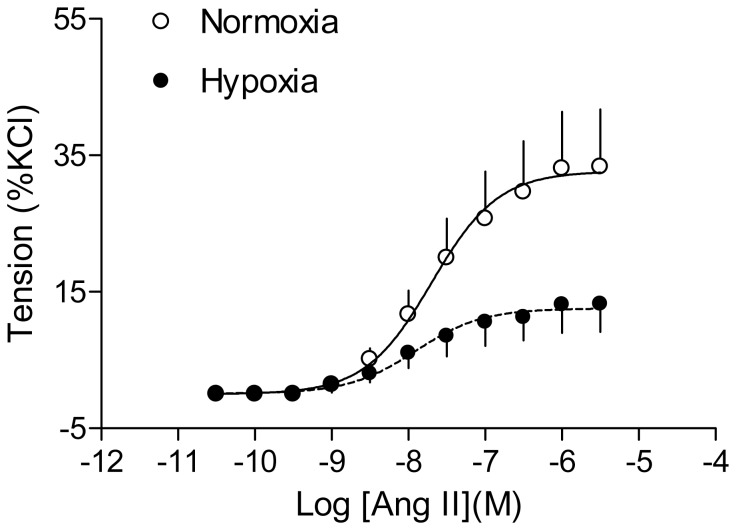
Effect of antenatal hypoxia on angiotensin II-induced arterial contractions in female offspring. Angiotensin II (Ang II)-induced contractions were determined in isolated aortas of adult female offspring that had been exposed *in utero* to normoxia or hypoxia. Data are means ± SEM, n = 9-10. The values of pD_2_ and the maximal response were presented in the text.

Ang II produced a time-dependent increase in arterial blood pressure in both control and hypoxia-treated adult offspring. As shown in Figure 1, antenatal hypoxia significantly enhanced Ang II-stimulated systolic, diastolic and mean arterial blood pressure in male offspring, as compared with the normoxic control group. However, hypoxia significantly reduced Ang II-stimulated systolic, diastolic and mean arterial blood pressure in female offspring (Figure 2). The baroreflex sensitivity determined as the slope of heart rate/mean arterial pressure was not significantly different between control and hypoxic groups in female offspring (Figure 3). Consistent with the reduced blood pressure response in female offspring, Ang II-induced arterial contractions were also significantly decreased in hypoxic animals (Figure 4). In isolated aortas, KCl (120 mmol/L)-induced contractions were not significantly different between hypoxic and normoxic control groups (2.03±0.11 *vs*. 1.98±0.11 g; P>0.05) (data not shown), suggesting the lack of effect of antenatal hypoxia on electromechanic coupling and vascular contractility. As shown in Figure 4, antenatal hypoxia had no significant effect on the pD_2_ (–log EC50) values of angiotensin II-induced contractions, as compared with the control (7.9±0.3 *vs*. 7.7±0.2, P>0.05). However, angiotensin II-induced maximal contractions were significantly decreased in antenatal hypoxia-treated female offspring, as compared with the control group (32.7±3.0% *vs*. 12.6±1.4% of KCl responses, P<0.05).

### Effect of antenatal hypoxia on plasma estrogen levels

**Figure 5:**
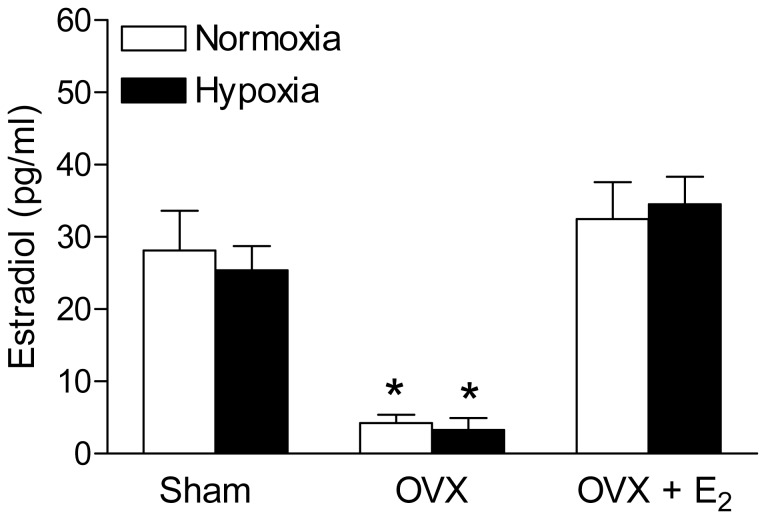
Effect of antenatal hypoxia on plasma estrogen level in female offspring. Blood plasma was collected from the both control and hypoxia-treated female offspring of sham, OVX and estrogen replacement groups. Plasma estrogen levels were determined with a commercially available Estradiol ELISA kit. Data are means ± SEM, n = 10–14 each group. * P<0.05, OVX vs. sham group.

Since our functional data indicated a gender difference in response to antenatal hypoxia, we hypothesized that alteration of estrogen level may play a key role in hypoxia-mediated changes of blood pressure response. We determined the plasma estrogen levels in different groups of female offspring. As shown in figure 5, antenatal hypoxia did not significantly changes plasma estrogen levels, as compared with the control of female offspring in sham, OVX and estrogen-replacement groups. OVX treatment significantly decreased plasma estrogen level, which was restored by estrogen-replacement in both control and hypoxic groups.

### Ovariectomy abrogated hypoxia-induced response in female offspring

**Figure 6:**
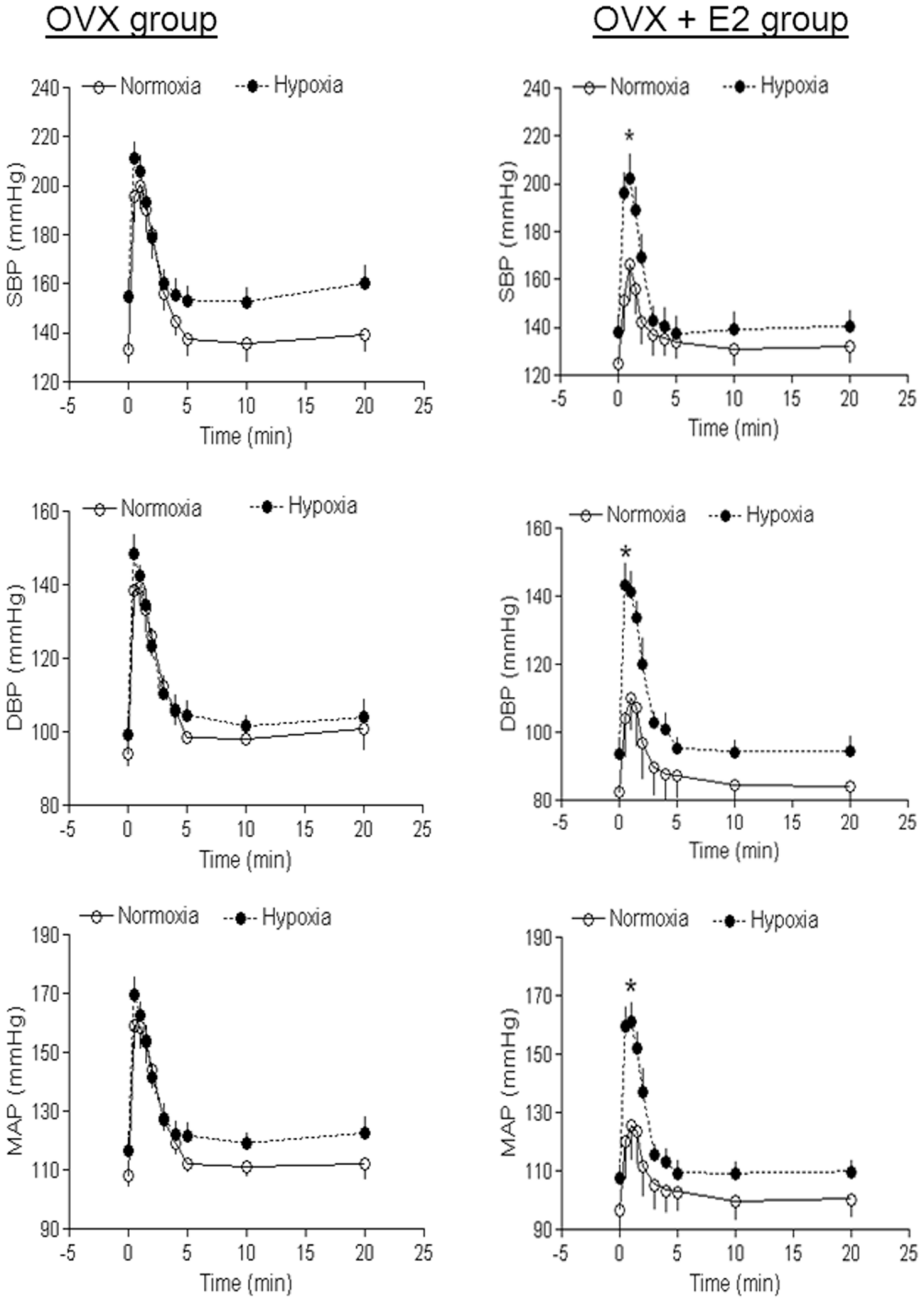
Effect of ovariectomy and estrogen replacement on angiotensin II-induced blood pressure response in female offspring. Systolic (SBP), diastolic (DBP), and mean (MAP) arterial blood pressure responses to angiotensin II (300 ng/kg; 1 ml/kg, *i.v.*) were measured in OVX female offspring (Left panel) and in OVX + E_2_ replacement (Right panel) that had been exposed *in utero* to normoxia or hypoxia. Data are means ± SEM, n = 7–9. * P<0.05, hypoxia *vs.* normoxia.

To determine whether ovarian function plays a key role in the antenatal hypoxia-induced effect in female offspring, Ang II-induced blood pressure response was determined in OVX animals. Unlike the findings in the sham animals, in OVX animals there was no significant difference in Ang II-induced blood pressure response between the control and hypoxic groups (Figure 6 left panel).

### Estrogen replacement did not rescue hypoxic effect

**Figure 7:**
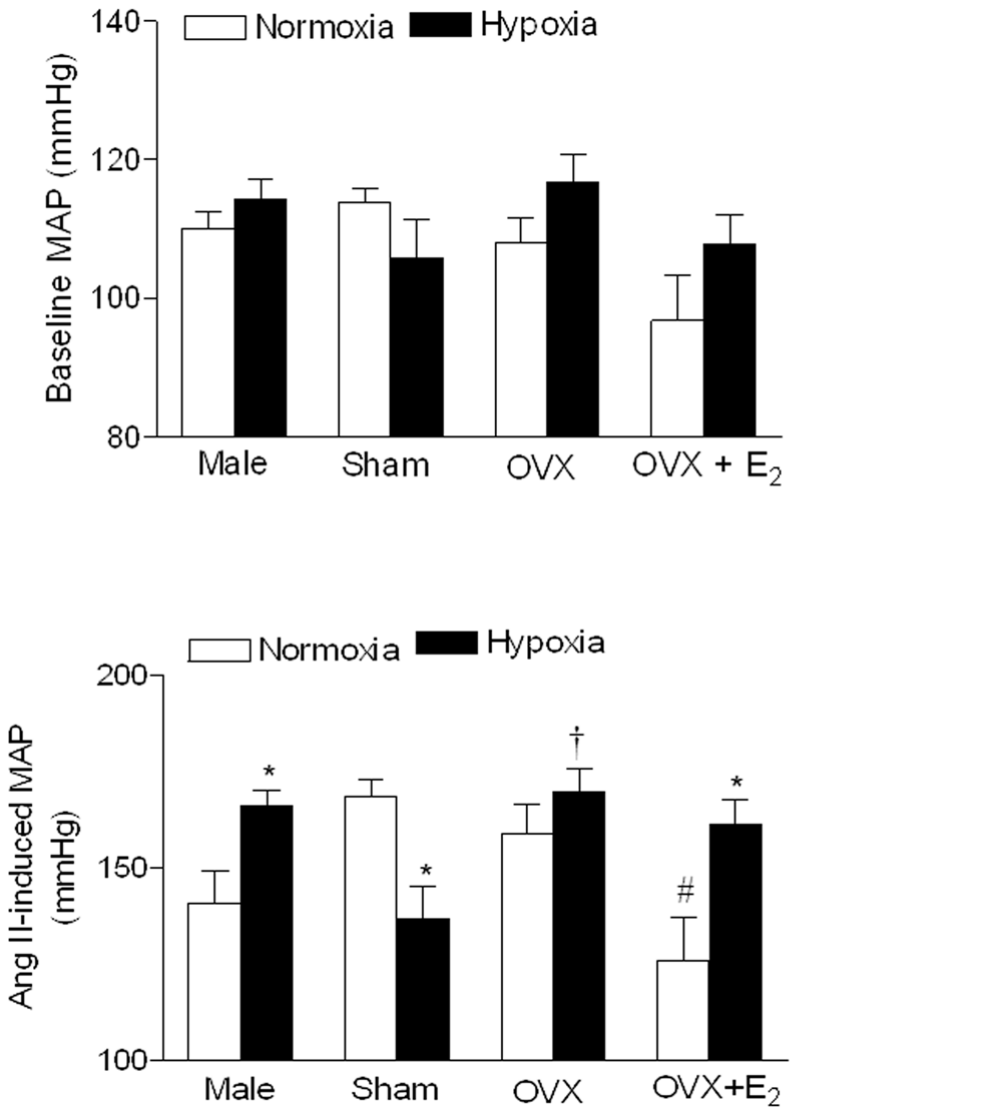
Effect of antenatal hypoxia on baseline and Angiotensin II-induced maximal mean arterial blood pressure responses in adult offspring. The baseline and maximal mean arterial blood pressure (MAP) responses to angiotensin II (300 ng/kg; 1 ml/kg, *i.v*.) were determined in adult male, sham, ovariectomy (OVX), and OVX plus estrogen (E_2_) replacement female offspring that had been exposed *in utero* to normoxia (control) or hypoxia. Data are means ± SEM, n = 7–9. * P<0.05, hypoxia *vs.* control; ^†^ P<0.05, OVX *vs*. sham; ^#^ P<0.05, OVX+E_2_ *vs*. OVX.

To further determine the role of estrogen in antenatal hypoxia-mediated blood pressure response, Ang II-induced blood pressure response was measured in OVX animals with estrogen replacement. In contrast to the findings in the sham animals, Ang II-induced blood pressure response was significantly greater in hypoxic than in control groups in OVX animals with estrogen replacement (Figure 6 right panel). In male offspring, the basal heart rate (Table 1) and mean arterial blood pressure (Figure 7) was not significantly different between control and hypoxic offspring. Similarly, in female offspring hypoxia also did not alter basal heart rate and mean arterial blood pressure. As shown in Figure 7, OVX had no significant effect on Ang II-induced blood pressure response in normoxic OVX as compared with normoxic intact animals, but significantly increased it in hypoxic OVX as compared with hypoxic intact animals and eliminated the difference between the control and antenatal hypoxic female offspring seen in the sham animals. Compared with OVX animals, estrogen replacement caused a significant decrease in angiotensin II-mediated response in normoxic control animals, but not in antenatal hypoxic animals, resulting in a reversal of blood pressure responses seen in the sham animals.

## Discussion

The present study has demonstrated a novel finding in a rat model that gestational hypoxia results in alteration of stress-induced BP response in adult female offspring. The major novel findings are as follows: (1) antenatal hypoxia resulted in a decrease in arterial blood pressure response to angiotensin II, which was not mediated by a change in the baroreflex sensitivity; (2) OVX had no significant effect on angiotensin II-induced blood pressure response in normoxic control animals, but significantly increased it in hypoxic animals and eliminated the difference between the control and antenatal hypoxic female offspring seen in the sham animals; (3) estrogen replacement caused a significant decrease in angiotensin II-mediated response in normoxic control animals, but not in antenatal hypoxic animals resulting in a reversal of blood pressure responses seen in the sham animals.

In present studies, we did not monitor the maternal food and water consumption, but previous studies in a similar hypoxic animal model have demonstrated that hypoxia reduces maternal food consumption [13]. Furthermore, Hemmings at al [13] in a paired food study have shown that maternal hypoxia treatment induces vascular alterations in adult offspring that are independent of the effects of nutrient restriction alone. These studies suggest that hypoxia may be directly responsible for the functional deficits reported. From our current study we expect that hypoxia may play a major role in responsible for the programming of vascular dysfunction, but we can not exclude the possible confounding effect of hypoxia and food intake on BP and vascular dysfunction. In the present study, we found that maternal hypoxia significantly decreased the body weights of near term fetuses but not the body weights of adult offspring, which are consistent with the previous studies [18], [32], [33]. These findings suggest an animal model of fetal stress-induced intrauterine growth retardation with a catch-up growth in the postnatal development. In addition, our current data also indicated that maternal hypoxia treatment did not affect the body weight of adult female offspring in sham control, OVX and OVX + E_2_ groups. However, 3 months after OVX treatment, the OVX rats had significantly higher body weights than the sham groups, suggesting that ovaries play an important role in regulation of female body weight. Similar findings have been reported in previous studies [34], [35]. EI-Seweidy et al [34] have also demonstrated that OVX-mediated increased body weights can be reversed by 6 weeks estrogen replacement treatment. However, our data indicated that 3-months estrogen replacement treatment not only reversed OVX-mediated increased body weights but also further lowered the body weights as compared with the sham group. Our findings suggest that estrogen may be one of the major factors released from ovary responsible for the down-regulation of female body weight, but other unknown factors released from ovary may partly counteract the effect of estrogen.

The finding that antenatal hypoxia had no significant effect on blood pressure under resting condition in adult offspring is in agreement with findings in several different animal models [18], [19], [32], [33]. Furthermore, the present finding in our animal model that antenatal hypoxia had no effect on basal BP but enhanced the BP response under agonist (Ang II) stimulation in adult male offspring, is consistent with Peyronnet et al [18] reported that although prenatal hypoxia did not affect cardiac parameters and blood pressure under resting condition, but significantly increased blood pressure in adult male rat offspring under stress stimulation. Similarly, our recent studies demonstrated that gestational hypoxia had no effect on baseline heart contractile function but significantly increased heart vulnerability to ischemia and reperfusion injury in male offspring [12]. In addition, previous studies in other animal models have also shown that intrauterine adverse environment has no effect on blood pressure under resting condition but significantly enhances blood pressure responses by agonist stimulation [32], [33]. These findings suggest that mild fetal stress may lead to sub-clinically altered cardiovascular function and increase the risk of cardiovascular dysfunction in offspring under stress stimulation. To our surprise, in contrast to the findings in adult male offspring with increased blood pressure in response to prenatal hypoxia, the present study demonstrated a significant decrease in arterial blood pressure response in females resulting from antenatal hypoxia. Similar findings of lowered blood pressure have been reported in several intrauterine malnutrition animal models [11], [36], [37]. While these findings may not support the hypothesis that all models of restricted fetal growth lead to hypertension later in life, whether this reduced blood pressure response represents a “good” adaptation is debatable given that it is a perturbation from the normal physiology. It is likely that the effects of perturbations on postnatal arterial blood pressure vary depending on the nature of intrauterine environment, species, gender difference, and postnatal pathophysiological conditions.

The gender differences of fetal programming of cardiovascular dysfunction in offspring have been reported in many different animal models [12], [14], [15], [32], [33], [38]–[40]. However, the mechanisms underlying the gender difference are not fully understood. Most of the studies indicate that male offspring develop hypertension, whereas female offspring seem to be protected [12], [14], [33], [40]. In the present study, we demonstrated that OVX abrogated the effect of hypoxia, suggesting a key role of ovarian function in antenatal hypoxia-mediated programming of blood pressure response in female offspring. The finding of a lack of effect of ovariectomy on blood pressure response in normoxic control animals in the present study is intriguing. Although it is generally accepted that ovary-released estrogen may lower arterial blood pressure, the interaction between ovarian hormones and arterial blood pressure is far more complex and controversial [15]. Previous studies showed that ovariectomy increased blood pressure while estrogen treatment reduced hypertension [41]. Conversely, other studies failed to show an effect of ovariectomy on blood pressure in female spontaneously hypertensive rats or normotensive rats [42], [43]. Thus, it is possible that other steroid hormones such as progesterone and/or other unknown factors released from ovary may counteract the effect of estrogen on the cardiovascular system. In support of this notion, it has been demonstrated that progesterone administration antagonizes the effect of estrogen on vascular function in women [44]. Unlike the findings in normoxic control animals, ovariectomy significantly increased angiotensin II-induced blood pressure response in hypoxic animals and eliminated the difference in blood pressure response between the control and hypoxic animals. This finding suggests that antenatal hypoxia results in reprogramming of ovarian function in female offspring. Indeed, it has been demonstrated in several animal models that *in utero* adverse environment may lead to reprogramming of ovarian function and alter ovarian steroid hormones release in adulthood [23]–[26]. In present study, we found that there were no differences of plasma estrogen levels between control and hypoxia-treated offspring. Consistent with current finding, previous studies also indicated that in utero adverse environmental exposure did not affect the circulating estrogen levels in adult females offspring [14], [30]. These findings suggest that the role of ovarian function in the regulation of cardiovascular function may be not only dependent on estrogen levels per se, but also on its effect on downstream signaling or the ratio of estrogen to other steroid hormones/factors-released by ovary. This can shift the vasoconstrictor-vasodilator balance of the ovary system, resulting in adaptive protection against hypertension in female offspring.

**Figure 8:**
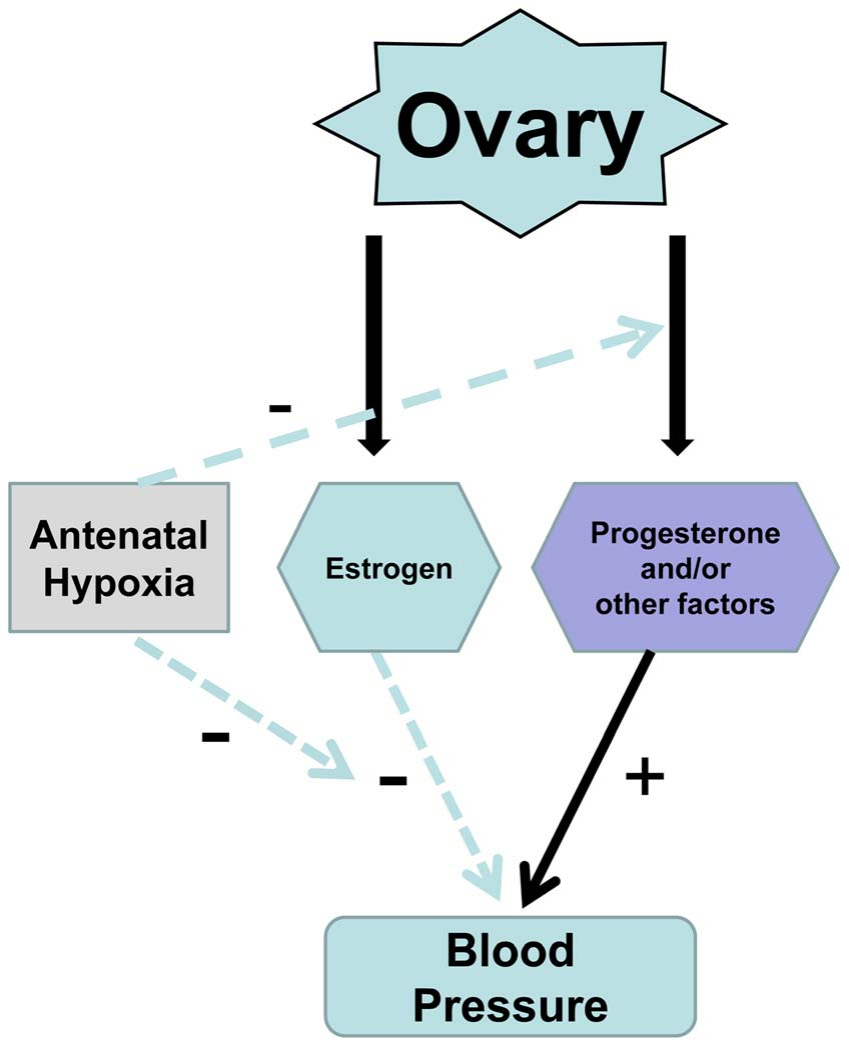
Potential mechanisms underlying fetal hypoxia-induced hypotensive response in female offspring. The ovary releases estrogen and other ovarian hormones/factors. Estrogen down-regulates arterial blood pressure, but other ovarian factors may counteract the effect of estrogen. Antenatal hypoxia leads to programming of a predominating decrease of ovarian progesterone/or other factors release but down-regulation of vascular estrogen effect, overall resulting in development of hypotensive reactivity in female offspring.

The present study demonstrated further that estrogen replacement in OVX animals decreased blood pressure response in normoxic control females. This supports the notion that estrogen plays an important role in regulating arterial blood pressure and in the protection of females from development of hypertensive reactivity. The finding that estrogen replacement in OVX animals had no significant effect on angiotensin II-mediated blood pressure response in antenatal hypoxic animals is very intriguing, and suggests a complexity of mechanisms in hypoxia-induced programming of ovarian steroid hormone function in female offspring. As shown in the diagram of Figure 8, antenatal hypoxia may cause a decreased ovarian progesterone/or other unknown factors release, leading to increased the relative ratio of estrogen to progesterone/other factor and shifted the balance of vasoconstriction/vasodilation, resulting in development of hypotensive reactivity in female offspring. Thus, in sham animals the effect of decreased ovarian steroid hormone other than estrogen release predominates with the phenotypic response of reduced blood pressure, which was abrogated in OVX animals. In OVX animals, the diminished effect of estrogen replacement on blood pressure response in hypoxic animals is likely to attribute to antenatal hypoxia-mediated down-regulation of estrogen-mediated vascular response. Indeed, the previous study of intrauterine growth restriction animal model demonstrated a significant decrease in vascular expression of estrogen receptors in offspring [45].

## Perspectives

Large epidemiological and animal studies indicate a link between *in utero* adverse stimuli during gestation and an increased risk of cardiovascular disease later in life. Gestational hypoxia is one of the most important and clinically relevant stresses to the fetal development. The present investigation shows a gender-dependent programming of blood pressure response in adult offspring and provides novel evidence of a complex mechanism in antenatal hypoxia-mediated programming of ovarian steroid hormone function in regulating vascular and blood pressure response in female offspring. Although the findings of the present study may not support the hypothesis that all models of fetal stress and intrauterine growth restriction lead to programming of hypertension later in life, whether the phenotype of reduced blood pressure response in female offspring really represents a “good” adaptation is certainly debatable given that it is a perturbation from the normal physiology. The complexity of mechanisms in fetal stress-mediated developmental programming of ovarian steroid function and vascular response warrants further investigation of its pathophysiological significance in the understanding of cardiovascular disease evolvement in females.
